# Dental Matters in Iowa

**Published:** 1863-10

**Authors:** Jas. Taylor


### Correspondence.

### DENTAL MATTERS IN IOWA.

Dear Register:—I have in my own mind, many times promised to write you, and tell you about matters in your chosen profession here in the "far West," but for various reasons have delayed from time to time doing so; and now if your readers will excuse all errors, I will undertake to fulfill my promise. I will first tell you, that your monthly visits to us "out here" are very precious and valuable, and when you fail to come at your regular times (as you sometimes do), we feel very anxious and disappointed; but soon you come loaded with good and profitable news, which shows that you have good and able fathers, and that they spare no pains in making you in every way worthy your name.

But now for dental matters. We dentists in Iowa are troubled with the same disease that dentists are in other States; viz., PROFESSIONAL JEALOUSY. Some have it *very bad*, so bad, that it makes them call their neighbor dentists *hard* and *ungentlemanly* names, when there is (in many cases) no other cause, but the effects of this terrible *plague*.

To get rid of this troublesome *monster disease* (and for other good objects too), we made a great effort last spring to get Iowa dentists together, and organize a Dental Association, and thus try whether we could not as an organized body, get ahead of this plague, and then do each other and our calling some good, as dentists are doing in your city and State, and in other cities and States I could mention. We met with great opposition in many ways and for many reasons, some (who of course had this disease) said, this matter should be started by OLD PIONEERS alone, and YOUNG PIONEERS should be like the boy who failed to learn his lesson, seen but not heard;—others who had the "plague" the most, thought that some of these prime movers in this matter only wanted to be popular, or perhaps wanted to be sent as delegates to the "National Dental Association," etc. But finally, a meeting was had at Muscatine, Iowa, in July last, and after a great deal of opposition a motion was passed, organizing a "State Dental Association." Then adopted a Constitution, elected officers, etc., and after some very interesting and profitable discussions and talk on various subjects, adjourned, promising all to be at our next meeting (which is soon to be held), and also feeling that "to know each other is to help each other." Now we want you and your friends everywhere to help us in this noble work. Our Secretary is Dr. A. J. McGarvey, of De Witt, Iowa,—let all who will, write to him a letter of encouragement, and let all who can come to our meeting—especially tell all *Iowa* Dentists to be *sure and be there.*

Our Secretary will tell you the time and place of meeting. We shall remember "Niagara Falls," and "Detroit," next year. More anon. WM. O. KULP.

MUSCATINE, Iowa, Sept. 18, 1863.

---

MESSRS. EDITORS:—In resuming my pen as a contributor again for the *Register*, allow me to apologize for my long silence, for I feel that I am addressing my old friends, many of whom have been constant readers of your valuable periodical from its commencement. When I relinquished the Editorship of the *Register*, I was in hope that a very few months would suffice to recuperate exhausted powers, and enable me to resume my pen at least, as *contributor*, with renewed vigor; but such was not the case, and I even now find that the very lazy habit of not writing at all, and which I have condemned so much in others, has almost mastered me. But now with renewed health, and as I trust, with renewed zeal in the cause of Dental education, I feel that I can no longer remain silent. Allow me then, without further preliminary, to report through the *Register* for the benefit of the profession at large, the result of a recent trip among the brethren.

Many of the profession are aware that in the effort to build up a Dental College in this city, a debt of four thousand dollars was left against the property. This debt by the liberality of the stockholders has been reduced to just four thousand dollars, to secure which, two mortgages have been given. The term for which this money was obtained having expired, it was decided to appeal to the liberality of the profession, to help extinguish the debt and place the institution on a firm basis for future usefulness. The Dental Society at St. Louis having been made acquainted with these facts, voluntarily resolved to aid in this matter, and invited me to their city for consultation on the subject.

On the 7th of the present month I left on this errand, and according to arrangement met the society on the evening of the 8th, at the house of Dr. I. Forbes, who, I am sorry to say, has been laid aside from professional labor for several months by an injury to his shoulder: we hope, however, ere this he is able to dispense his skill to his many friends.

I had the pleasure of seeing a few of the alumni of the Institution at this meeting, Dr. C. W. Spaulding being the presiding officer, and Dr. A. M. Leslie, Secretary; also, Drs. McKellops and Peebles being present—all these gentlemen showed that they had not forgotten their Alma Mater, nor indeed their profession.

I have often expressed my high appreciation of the profession in St. Lous, but when I landed in their city, after the most unpleasant night's ride I ever had, and met the hearty welcome, the warm and generous greeting which was on every occasion extended me, the chill and discomfort of the journey passed as a fleeting cloud, and all my professional enthusiasm (if I ever had any,) came back upon me with redoubled force. It has done me good, and I feel more proud of my profession than ever before.

I wish to return my sincere thanks to all the members of the profession at St. Louis that I met, for their courtesy, and especially our former townsman, Dr. A. M. Leslie, for his unwearied kindness and personal exertion on behalf of the College. I regret exceedingly that time did not permit me to call on more of the profession in St. Louis, and to visit more of the beautiful city and its environs.

On Friday evening the 9th, I left for Chicâgo, with many warm commendations to the brethren of that city; urging upon them, that as they claim to be the larger city, so should their liberality more largely abound. The list of subscribers and the amount of stock taken at both places, will show how well they responded to the call. In justice to the profession at Chicago I must, however, say that I hope still, when I hear from one of our alumni of that city, who I had not the pleasure of meeting, their liberality will equal that of St.

Louis. Yet I have again to refer to the latter place—for our friend, Dr. C. W. Spaulding, who never sleeps in a good cause, and who loves the profession with a noble love, sends me the name of another subscriber—which makes fourteen shares for them. Now Messrs. Editors, since my return I have secured two additional shares in our own city—can not we now hope that our Institution will soon be free from debt, and even more, this subscription be extended to five thousand dollars, leaving one thousand for anatomical specimens, chemical apparatus, etc., etc.

It will be observed that the profession at St. Louis, as well as Chicago, have subscribed on condition that enough stock is taken to pay off the entire debt, and that the stock be non-interest paying. The object of this was to relieve the Teachers in the College from a heavy tax which they felt should not be imposed upon them, and so that ultimately a fund could be set aside for Library, etc., etc. I must say that in all my intercourse with the profession while away, I met with the most high-minded and liberal views of Dental education; and I can justly say, that only in two or at most three instances did I make application for stock, but that it was cheerfully taken. In Chicago, for want of a local society, my labor was far greater than it would have been, and I have no doubt at all, if they had had an efficient society there, I should have sent back to St. Louis a much larger report. I do not, however, regard the amount of stock taken in either place as all which might be procured; for you will observe that not half the profession of either place have as yet been called on. I hope ere this, the brethren of Chicago have formed a society for their own protection and benefit. I am sure they have the material for such an organization, and there is not a practitioner in the city but would be benefited thereby.

Much, very much professional *slang*, if traced out would lie at the door of *shopping patients*, who are specially interested in getting a reduction of *fees*, who in their zeal to reduce fees often forget that truth is of more value than even gold.

Let me assure the profession, a few determined high-minded men in any city *can* eradicate this evil, and in doing this they will rid themselves of a great nuisance, and benefit not only the profession, but the public.

I might, if I had time, speak of the great good of our Dental Associations. Suffice it to say, that I have found that those who attend these Associations are generally fully awake to the honor of the profession, and readily contribute of their means for its advancement.

In Chicago, for instance, I met one of the profession whose acquaintance I had formed at New York, at the meeting of the Dental Convention. I allude to Dr. Abell, who will excuse me for introducing his name in this connection. On entering his office, I was surprised to be at once recognized and most cordially welcomed; and as soon as my business was made known, he remarked that the object was worthy of aid, and only hesitated to take two shares, because none others in that city had taken more than one; but when told that Dr. Allport would take two, he at once subscribed the like number. Dr. Allport having visited our Institution was posted up in the matter, and was ready and willing to do as much as any other. Chicago claims some excellent operators, and I hope when I visit them again, to meet them as a Fraternity of Brethren ; regularly organized, working together for the good of the profession.

Time and space will not allow me to scarcely allude to half of the kindness and attention bestowed. I must attribute far the greater part of it to that professional pride for which Dentistry is becoming noted. There appears to be a disposition, increasing more and more, to take full rank side by side with our Mother Science ; feeling that dental science is a mere specialty of Medicine and Surgery, and that the like facilities must be secured for our students which is afforded in Medical Schools.

I append the subscription paper with the names of those who have taken stock since my visit to St. Louis, and the number of shares subscribed by each; remarking for the benefit of those who may wish their names added to this list, that those who take two shares of stock, pay one hundred dollars on or before the 20th of February next, and for the second share of stock, one hundred a year from that date.

We feel assured that there are many others who would like to have their names handed down as founders of the first College ever erected exclusively for the benefit of Dental education. We solicit their names, hoping to meet with a response from many whom we shall be unable to visit or perhaps even write to.

The following is the subscription paper gotten up by the St. Louis Dental Society, through the committee, Drs. C. W. Spaulding, and A. M. Leslie.

"We the undersigned, desiring to see dental education established in the West on a permanent and independent financial basis, agree to take stock in the Ohio Dental College Association as per subscription below, on the following conditions, viz.:—

1st. Sufficent new stock must be subscribed, to provide the means necessary to pay off the debt now secured by mortgage on the College building and lot belonging to said Association.

2d. The present and future stockholders shall and do agree to make the whole stock of said Association a non-interest paying stock.

3d. The College building shall be used for the highest purposes of dental education, through a regular Faculty, and the property shall not be diverted to other uses without the consent of two-thirds of the stockholders.

4th. In case the College building ceases to be used for the purposes first above mentioned, then after six months notice a majority of the stockholders may order a sale of the property of the Association, and the net proceeds of said sale shall be divided *pro rata* among the stockholders, or their legal representatives.

St. Louis, Oct. 9th, 1863.

SUBSCRIBERS.

| Names. | Residence. | No. of Shares. | Names. | Residence. | No. of Shares. |
|---|---|---|---|---|---|
| C. W. Spaulding, | St. Louis, | Two. | W. W. Allport, | Chicago, | Two. |
| Isaiah Forbes, | " | Two. | J. D. Quinlan, | " | One. |
| H. J. McKellops, | " | Two. | J. A. Kennicott, | " | One. |
| Aaron Blake, | " | Two. | Jno. C. Fuller, | " | One. |
| Henry Barron, | " | Two. | Emanuel Honsinger, | " | One. |
| A. D. Sloan, | " | Two. | T. P. Abell, | " | Two. |
| A. M. Leslie, | " | One. | Wm. Albaugh, | " | One. |
| Wm. N. Morrison, | " | One. | James C. Dean, | " | One. |
| S. T. Creighton, | Chicago, | One. | James Leslie, | Cincinnati, | One. |
| Geo. H. Cushing, | " | One. | B. D. Wheeler, | " | One. |

We hope soon to make the list stand fourteen for Chicago, the same as St. Louis.

JAS. TAYLOR.

